# Prevalence, Reasons, and Perceived Benefits of Complementary and Alternative Medicine Among Patients With Rheumatic Diseases in Morocco: A Monocentric Cross-Sectional Study

**DOI:** 10.7759/cureus.66015

**Published:** 2024-08-02

**Authors:** Fatine Kronbi, Latifa Tahiri, Sara Bahloul, Samya Ez-zaoui, Imane Bensaghir, Samia El Hilali, Jihane Belayachi, Redouane Abouqal, Hanan Rkain, Fadoua Allali

**Affiliations:** 1 Department of Rheumatology B, Ayachi Hospital, Ibn Sina Hospital Center, Faculty of Medicine and Pharmacy, Mohammed V University, Rabat, MAR; 2 Department of Rheumatology B, Sheikh Khalifa International University Hospital, Mohammed VI University of Health Sciences, Casablanca, MAR; 3 Laboratory of Biostatistics, Clinical, and Epidemiological Research, Faculty of Medicine and Pharmacy, Mohammed V University, Rabat, MAR; 4 Acute Medical Unit, Ibn Sina University Hospital, Rabat, MAR; 5 Exercise Physiology and Autonomous Nervous System Team, Laboratory of Physiology, Faculty of Medicine and Pharmacy, Mohammed V University, Rabat, MAR

**Keywords:** complementary and alternative medicine (cam), survey research, patients’ perceptions, cupping therapy, rheumatic diseases

## Abstract

Background

In general, rheumatologists often have limited knowledge regarding the use of complementary and alternative medicine (CAM) among patients with rheumatic diseases. Understanding the prevalence, reasons for use, and perceived benefits of CAM can help improve patient care and guide clinical practices. This study aimed to assess the prevalence, reasons for use, and perceived benefits of cupping therapy, apitherapy, and traditional cautery treatments among patients with rheumatic diseases. Additionally, it aimed to explore adverse effects, location and pricing disparities in CAM practices, information sources, and the rate of reporting CAM use to treat rheumatologists and to identify factors associated with the use of these CAM approaches.

Methodology

This single-center, cross-sectional study was conducted in a Moroccan University Hospital and included patients receiving care for rheumatic diseases during hospitalization or outpatient visits from January 2024 to March 2024. The data were collected using a structured, validated, and pilot-tested questionnaire administered by attending rheumatologists. The questionnaire aimed to gather demographic information and to identify patients using CAM, collecting data related to their utilization.

Results

A total of 100 patients were enrolled (mean age: 52.3 ± 12.8 years, 75% female). Among them, 43% had chronic inflammatory rheumatism. Additionally, 46% reported using at least one of the three CAM methods studied, with 36% using cupping therapy (specifically the wet method), 9% using apitherapy, and 16% using traditional cautery. The main reason for using CAM was to alleviate their pain (55%). Perceptions of effectiveness were reported by 38% of patients using cupping therapy, 20% using apitherapy, and 5.9% using traditional cautery. The use of CAM was significantly lower in patients with a university education (odds ratio = 0.05, 95% confidence interval = 0.003-0.92).

Conclusions

Our study revealed a significant prevalence of CAM use among patients with rheumatic diseases in Morocco, with cupping therapy emerging as the most commonly utilized method. These findings underscore the importance of enhancing awareness and understanding of CAM practices among both patients and healthcare providers to promote more structured and informed CAM practices.

## Introduction

Complementary and alternative medicine (CAM) or “complementary health approaches” are a group of diverse medical and healthcare practices and products that are not presently considered part of conventional medicine [1].

In recent years, there has been a notable surge in interest surrounding CAM, including therapies such as cupping therapy, apitherapy, and traditional cautery [2]. Cupping therapy is a therapeutic technique that utilizes a vacuum force created beneath a small vessel applied to the surface of the skin. This approach focuses on blood and autologous healing substances in a specific area, stimulating metabolic activity and improving immune function. Additionally, it aids in stabilizing blood biochemistry [3]. Apitherapy, known for its therapeutic use of beehive products since ancient times, is now beginning to reveal its mechanism of action [4]. Traditional cautery, which utilizes hot iron, involves creating burns on the tissue to either close wounds, stop bleeding by promoting blood clotting, or remove part of the body [5].

In Morocco, we observed an inclination among patients to use CAM to alleviate illnesses. However, the country lacks comprehensive studies on this subject, and many healthcare professionals may harbor negative or unscientific biases against these practices. Understanding the prevalence, reasons for use, and perceived benefits of CAM can help improve patient care and guide clinical practices.

Therefore, the primary objectives of our study were to assess the prevalence, reasons for use, and perceived benefits of cupping therapy, apitherapy, and traditional cautery treatments among patients with rheumatic diseases. Furthermore, it aimed to explore adverse effects, location and pricing disparities in CAM practices, information sources, and the rate of reporting CAM use to treating rheumatologists, and to identify demographic and clinical factors associated with the use of these CAM approaches.

## Materials and methods

Study type and population

We conducted a cross-sectional survey among patients receiving care for various rheumatic diseases at Al Ayachi University Hospital, a specialized rheumatology and physical medicine and rehabilitation facility located in Sale, Morocco. The study included patients with chronic inflammatory rheumatic diseases, such as rheumatoid arthritis (RA) and ankylosing spondylitis (AS), as well as degenerative joint diseases, such as lumbar radiculopathy and osteoarthritis. The data collection period spanned from January 2024 to March 2024.

Study eligibility criteria

Participants had to meet the following eligibility criteria: be over 18 years old, fluent in dialectal Arabic, and either hospitalized or attending consultations for rheumatic diseases from January 2024 to March 2024. No specific exclusion criteria were applied for this study.

Sampling

A convenience sampling method was used. All patients with rheumatic diseases who presented for hospitalization or consultation in the rheumatology department between January 2024 and March 2024 and agreed to participate were included in the study. Consequently, sample size calculations were not performed in advance.

Data collection

A face-to-face interview was conducted using a structured and validated questionnaire administered by the rheumatology medical team. The questionnaire was developed by two rheumatologists after an extensive literature review of similar studies. It was then pilot-tested on five patients from the rheumatology department at the hospital.

During the pilot test, patients were asked to check for question understanding and language clarity. Their comments were used to simplify the questions as much as possible without affecting content accuracy. Data obtained in this pilot part of the study were omitted from the final analysis.

A comprehensive questionnaire with 20 revised questions was used to gather demographic and clinical information about the patients, including age, sex, occupation, education level, place of residence, monthly income, and details of their medical conditions. The questionnaire also aimed to identify CAM users and collect data on their utilization, including reasons for CAM use, specific types of CAM used, perceptions of effectiveness, adverse effects, perceptions of complications, costs per session, and locations of practices. Additionally, it gathered information on sources of CAM awareness, and whether patients informed healthcare providers about their CAM use, including reasons for not disclosing it.

Statistical analysis

The data were entered and analyzed by the Public Health Department of the Faculty of Medicine and Pharmacy in Rabat, and a descriptive analysis of the validated data was performed.

Descriptive statistics were used to summarize the demographic and clinical characteristics of the patients. Qualitative variables were expressed as numbers and percentages, and quantitative variables were expressed as mean and standard deviation if the distribution was Gaussian or as median with the interquartile range if the distribution was asymmetric (non-Gaussian).

Univariate analysis was performed using the chi-square test, Fisher’s exact test, and Mann-Whitney test, depending on the test conditions. Logistic regression analysis was performed to assess the independent effects of age, sex, level of education, and the presence of an inflammatory disease on the use of CAM.

A list of explanatory variables was established according to the results of the univariate analysis and the relevance of the variables. Differences were considered statistically significant if the p-value was less than 0.05. Associations were expressed as odds ratios (ORs) with 95% confidence intervals (CIs). Data analysis was performed using the Jamovi 2.3.19 statistical software and R version 4.3.1.

Ethical considerations

Ethical and regulatory aspects were taken into consideration before starting this study, in particular, the submission of the protocol to the Biomedical Research Ethics Committee, Faculty of Medicine and Pharmacy, Mohamed V University in Rabat, Morocco (approval number: 53/24) in accordance with the ethical standards of the 1964 Declaration of Helsinki and its later amendments or comparable standards. The participants were informed of the study aim and data collection process, as well as the voluntary nature of participation in the research. Informed consent was obtained from study participants, and the rules of anonymity were respected for each study participant.

## Results

Demographic and clinical characteristics

Of 170 eligible patients, 100 agreed to respond to the questionnaire, resulting in a response rate of 58.8%. The average age of the patients was 52.3 ± 12.8 years, and 75% were women. The majority of patients (84%) resided in urban areas, and 51% of them were illiterate. Almost half of the patients (52%) had individual monthly incomes below the guaranteed interprofessional minimum wage (SMIG), which is equivalent to 2,970.05 MAD or 270.94 euros.

Among the patients with rheumatic diseases, 43% had chronic inflammatory rheumatism, and 19% were on biotherapy. Patient demographics are shown in Table 1.

**Table 1:** Demographic and clinical data of the participants. *: Expressed as mean and standard deviation; **: Expressed as n (%). CAM = complementary and alternative medicine

Characteristics	CAM use			
	All patients (n = 100)	Used CAM (n = 46)	Never used CAM (n = 54)	P-value
Average age (years)*	52.3 ± 12.8	53.1 ± 12,9	51.4 ± 12.8	0.4
Gender**				0.7
Female	75 (75)	34 (73.9)	41 (75.9)	
Male	25 (25)	12 (26.1)	13 (24.1)	
Education level**				0.01
Illiterate	51 (51)	25(54.3)	26(48.1)	
Primary school	15 (15)	8 (17.4)	7 (13)	
Secondary school	9 (9)	6 (13)	3 (5.6)	
High school	10 (10)	5 (10.9)	5 (9.3)	
University studies	15 (15)	2 (4.3)	13 (24.1)	
Place of residence**				0.8
Urban area	84 (84)	39 (84.8)	45 (83.3)	
Rural area	16 (16)	7 (15.2)	9 (16.7)	
Nature of rheumatic disease**				0.2
Degenerative diseases	57 (57)	23 (50)	34 (63)	
Chronic inflammatory rheumatic diseases	43 (43)	23 (50)	20 (37)	
Rheumatoid arthritis	30 (30)	15 (32.6)	15 (27.8)	
Spondyloarthritis	13 (13)	8 (17.4)	5 (9.3)	
Patients on biotherapy**	19 (19)	10 (21.7)	9 (16.7)	0.9
Individual monthly income below the minimum wage**	52 (52)	37 (7.2)	36 (72)	0.8

Prevalence of different complementary and alternative medicine practices

Among our patients, 46% had previously tried at least one form of CAM. Notably, the most common CAM was cupping therapy (36%), followed by traditional cautery (16%) and apitherapy (9.9%).

**Figure 1:**
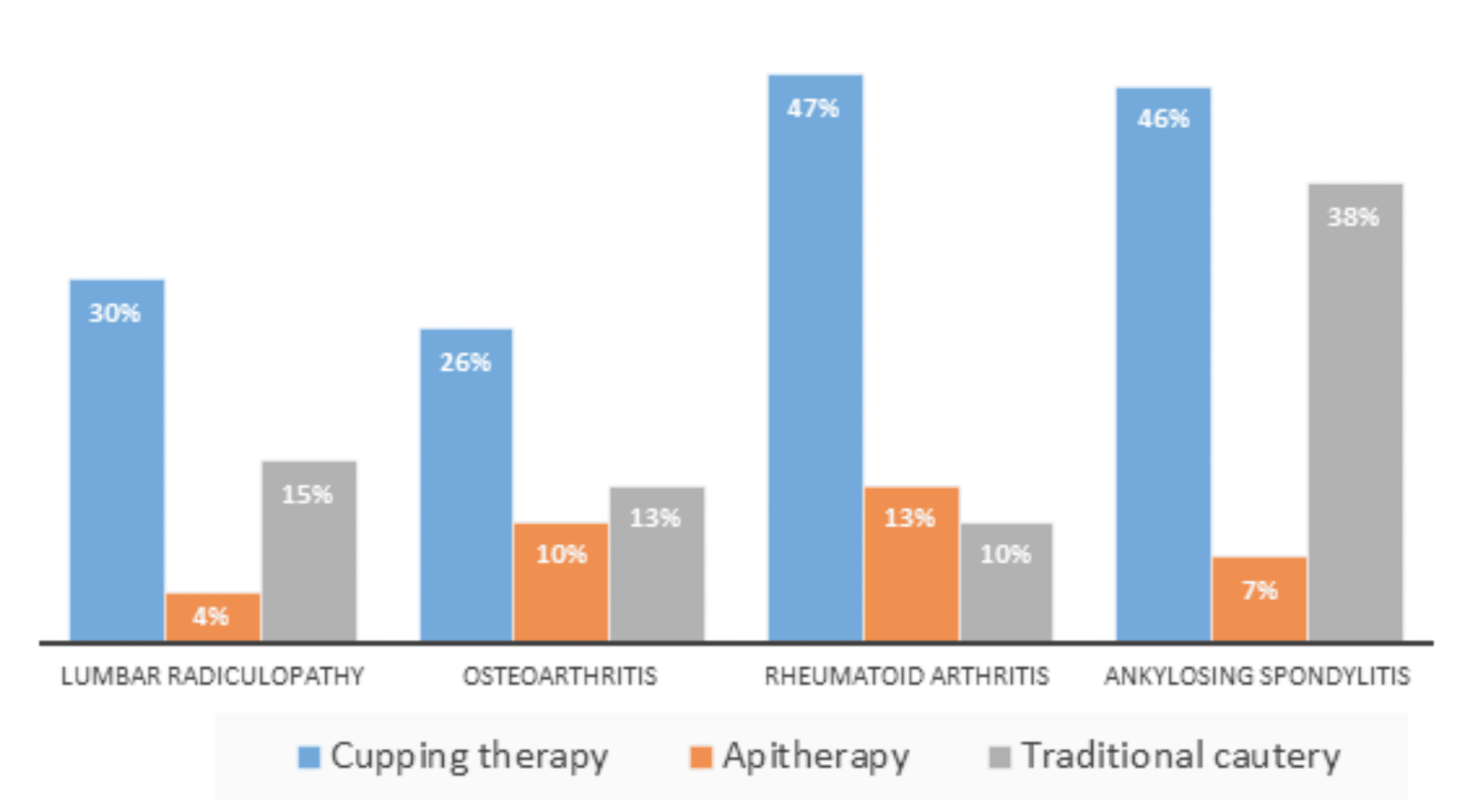
Prevalence of complementary and alternative medicine methods in each pathology.

When rheumatic diseases were divided into diagnostic subgroups, cupping therapy was particularly prevalent among patients with chronic inflammatory rheumatic diseases, with higher rates observed in RA (47%) and AS (46%) compared to degenerative joint diseases (30% in lumbar radiculopathy and 26% in osteoarthritis) (Figure 1).

Reasons for complementary and alternative medicine use

Stated reasons for CAM use were alleviation of pain for 65% of the patients, reducing physical fatigue for 43%, alleviating mental fatigue for 39%, curing illness for 21%, and its perceived cost-effectiveness compared to prescribed treatments for 10% of the patients.

Perceptions of the efficacy of complementary and alternative medicine methods

**Figure 2:**
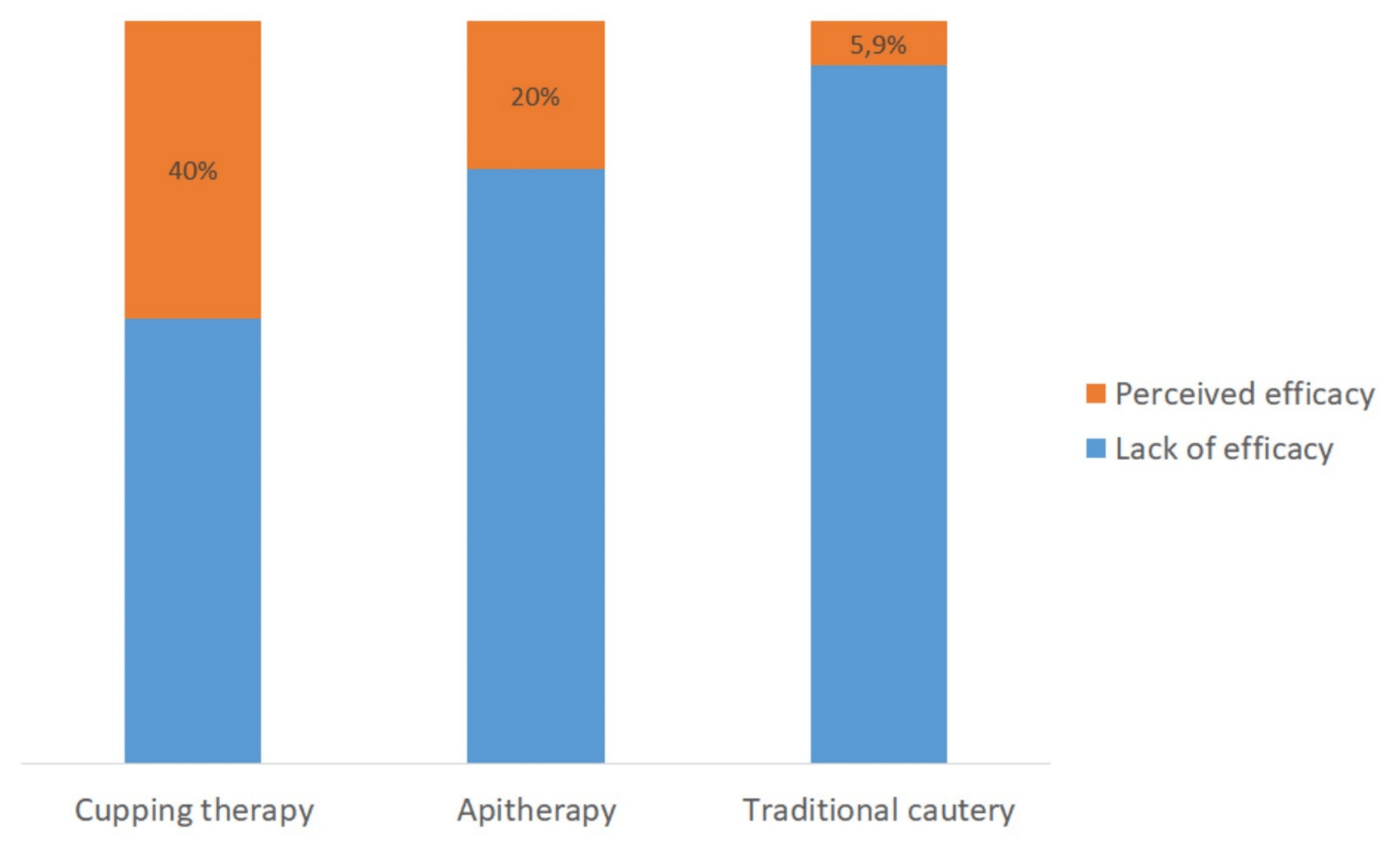
Perceived efficacy by patients of different complementary and alternative medicine practices.

Overall, 40% of patients who used cupping therapy perceived the efficacy of this method compared to apitherapy (20%) and traditional cautery (5.9%) (Figure 2).

Information source and rate of reporting the use of complementary and alternative medicine to treating rheumatologists

**Figure 3:**
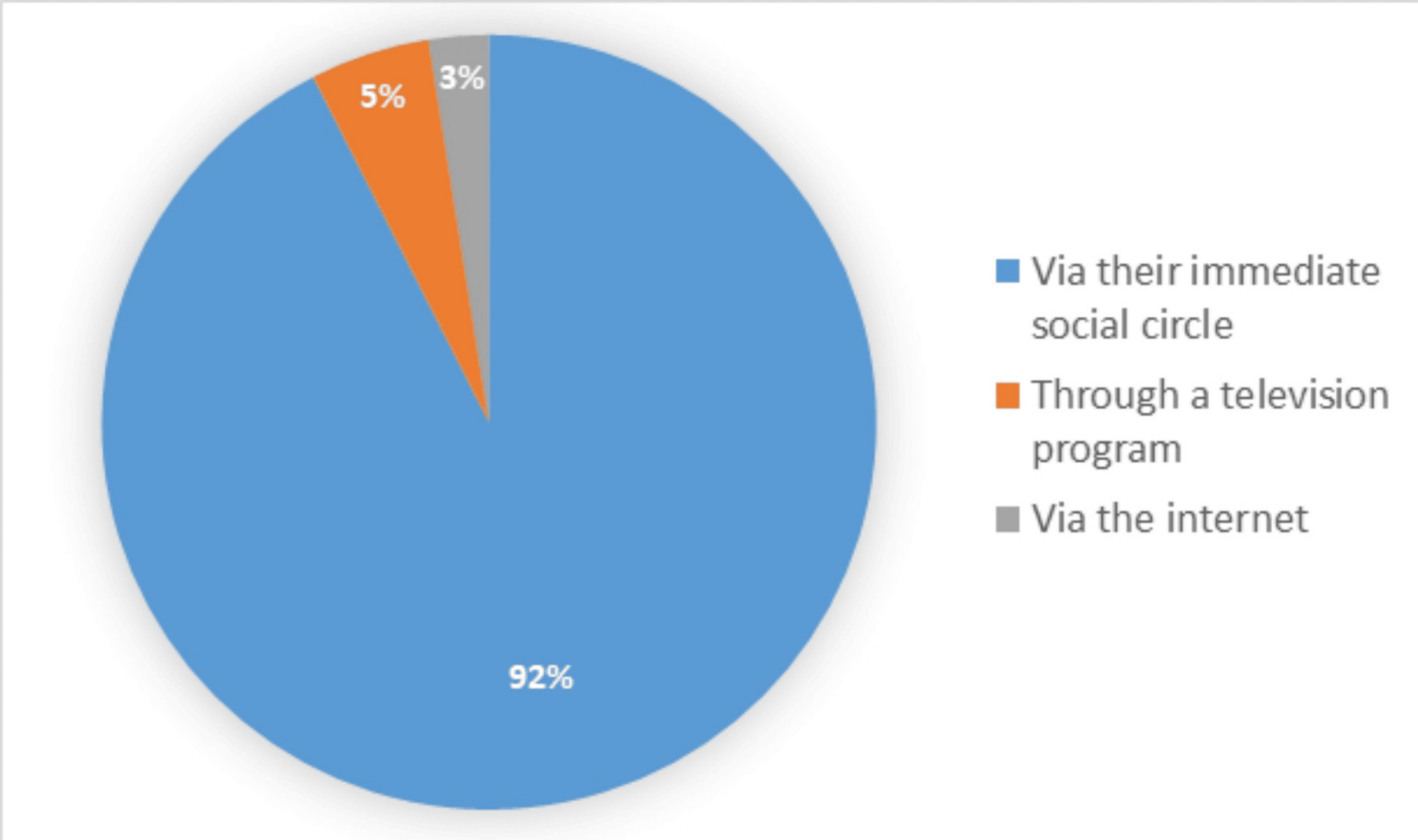
Source of information about complementary and alternative medicine practices.

Results showed that the predominant source of information from which they obtained knowledge for using CAM for 92% of patients was their immediate social circle, comprising family and friends (Figure 3).

**Figure 4:**
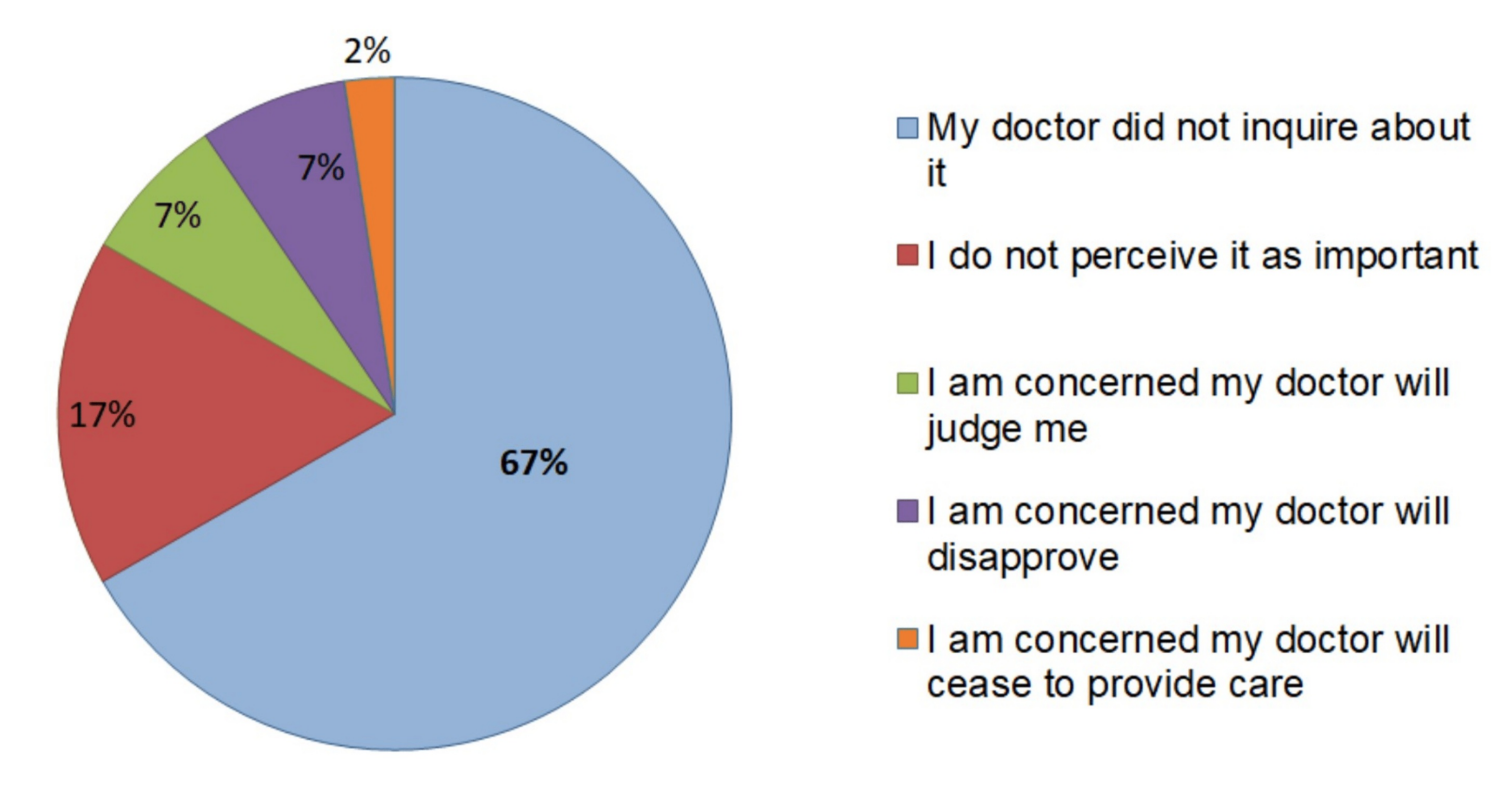
Reasons for non-disclosure of complementary and alternative medicine usage to doctors.

The majority of patients (91%) did not disclose their use of these alternative medicines to their attending rheumatologist, which was primarily attributed to the fact that their doctors did not inquire about it in 67% of cases (Figure 4).

Complementary and alternative medicine practice location

**Figure 5:**
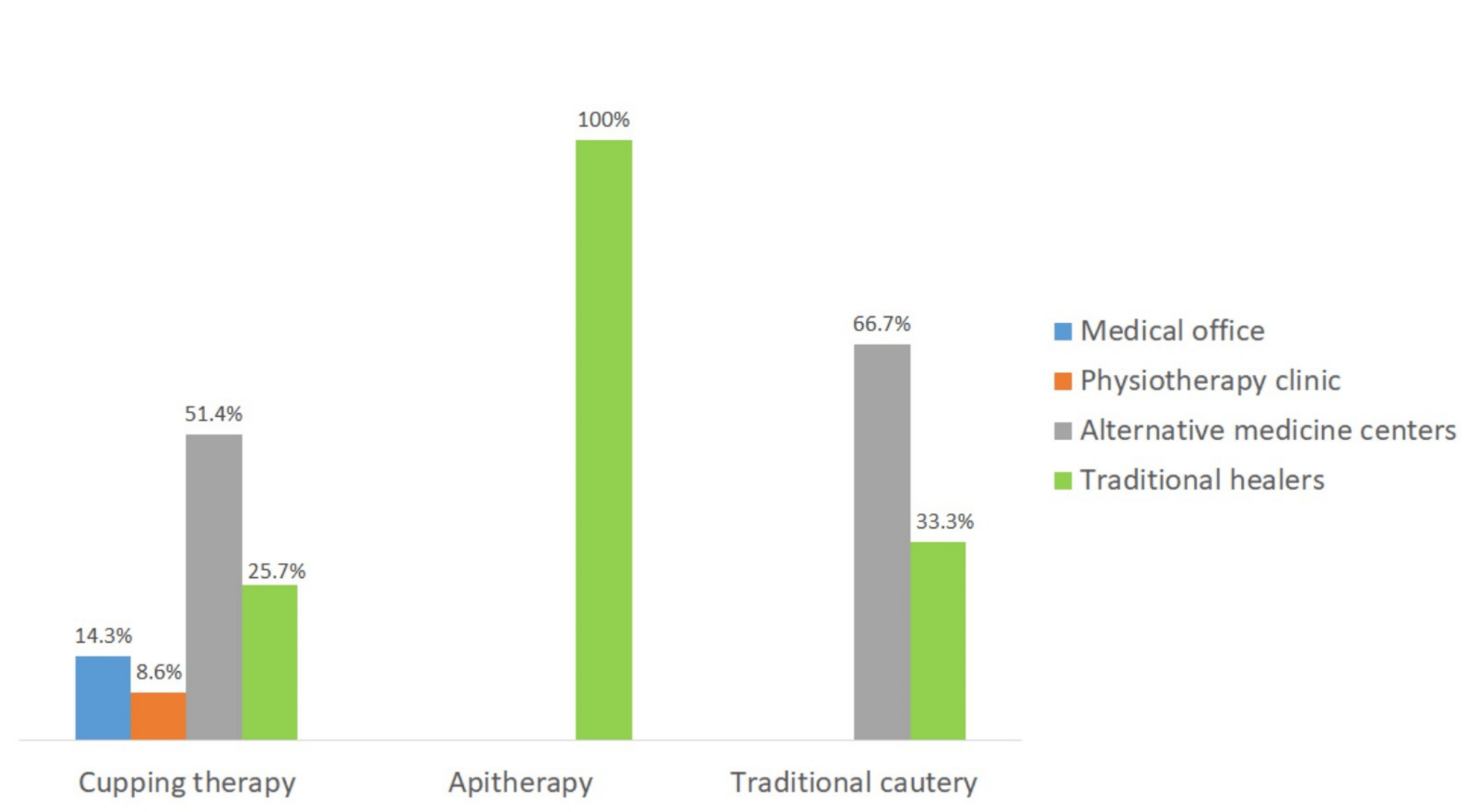
Locations of complementary and alternative medicine practices among patients.

Only 22.9% of patients received cupping therapy from medical or paramedical centers, typically provided by doctors or physiotherapists. In contrast, 77% of patients sought non-medical and non-paramedical therapeutic centers (alternative medicine centers) or traditional healers. Apitherapy was practiced in alternative medicine centers (66.7%) and provided by traditional healers (33.3%), while traditional cautery was solely performed by traditional healers (100%) (Figure 5).

Complications of complementary and alternative medicine use

Regarding adverse effects, 9% of the patients had reported reversible side effects linked to the CAM under study. Five patients reported local infections following traditional cautery, two patients experienced minor allergic reactions post-apitherapy, and one patient documented vertigo and tachycardia after cupping therapy. When considering patients’ perceptions of the frequency of adverse effects associated with the use of various alternative practices, 65% of patients believed that traditional cautery was most likely to cause adverse effects, followed by apitherapy (28% of patients). Cupping therapy was perceived as the least likely to cause adverse effects, with only 17% of patients attributing adverse effects to it.

Pricing disparities in complementary and alternative medicine practices

**Figure 6:**
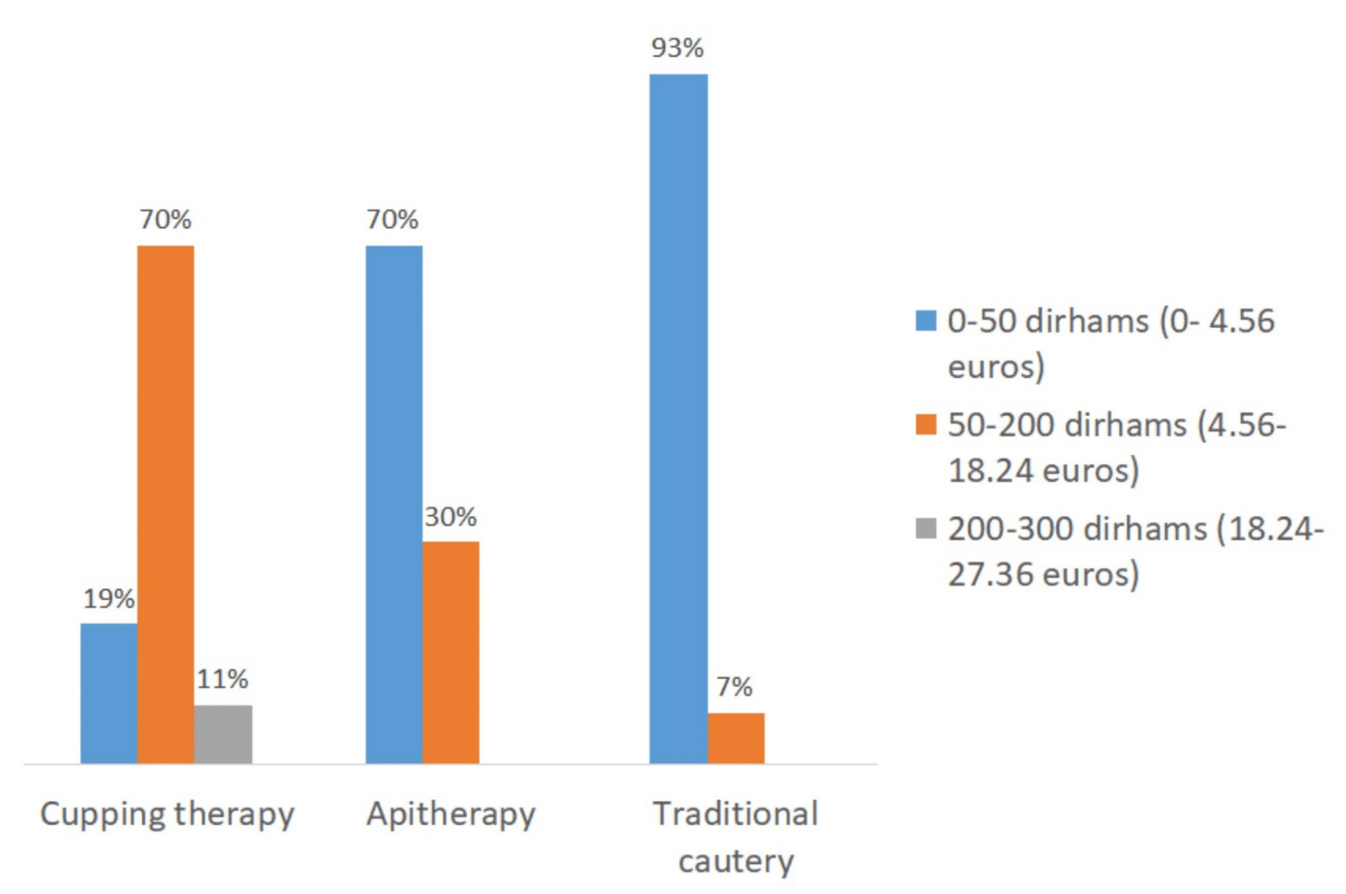
Cost of complementary and alternative medicine sessions.

Overall, 70% of patients reported paying between 50 and 200 dirhams (equivalent to 4.56 to 18.24 euros) per cupping therapy session, while 70% of patients practicing apitherapy and 93% of those practicing traditional cautery paid less than 50 dirhams (equivalent to 4.56 euros) per session (Figure 6).

**Figure 7:**
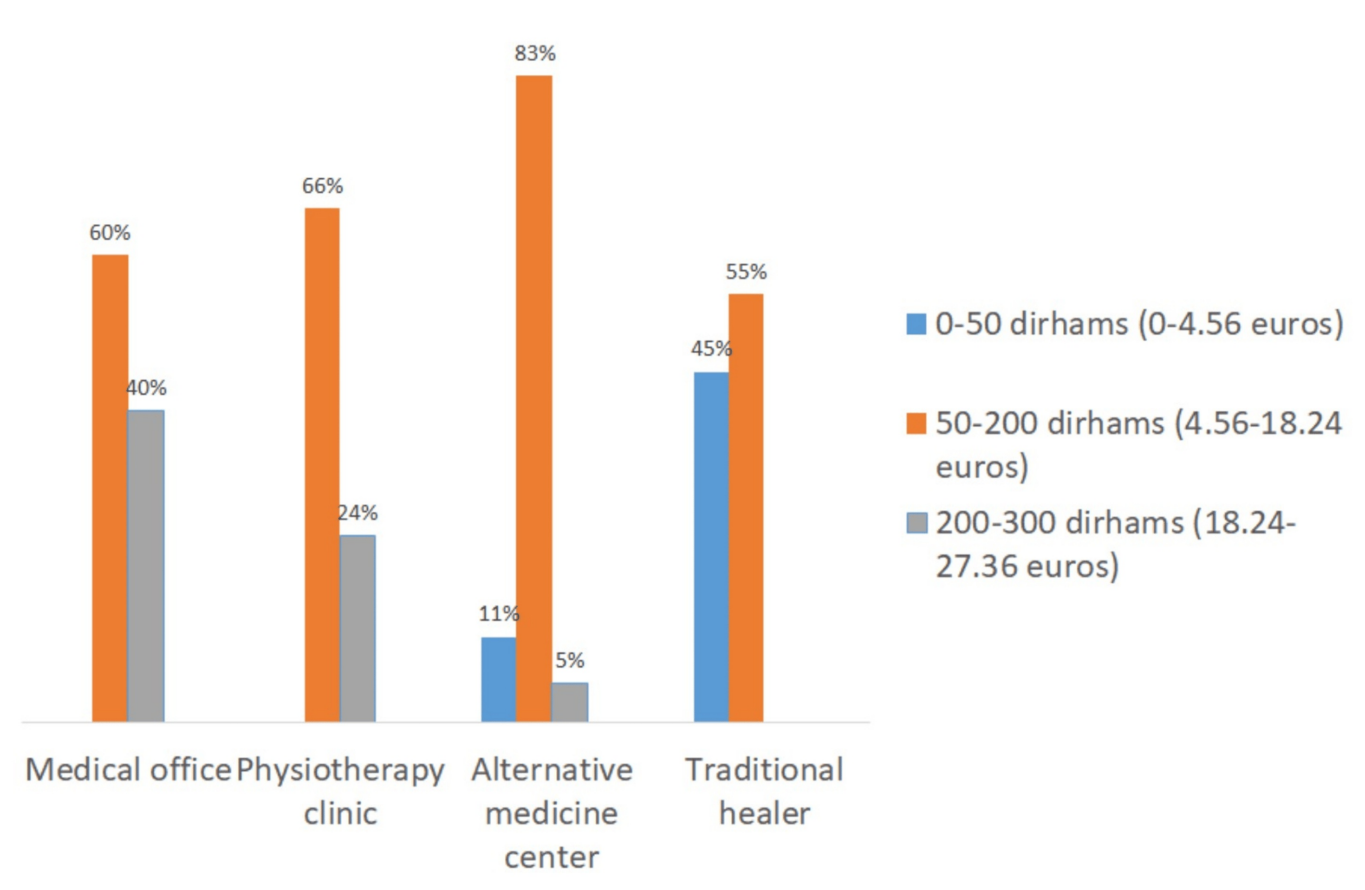
Cost of cupping therapy session according to practice location.

**Figure 8:**
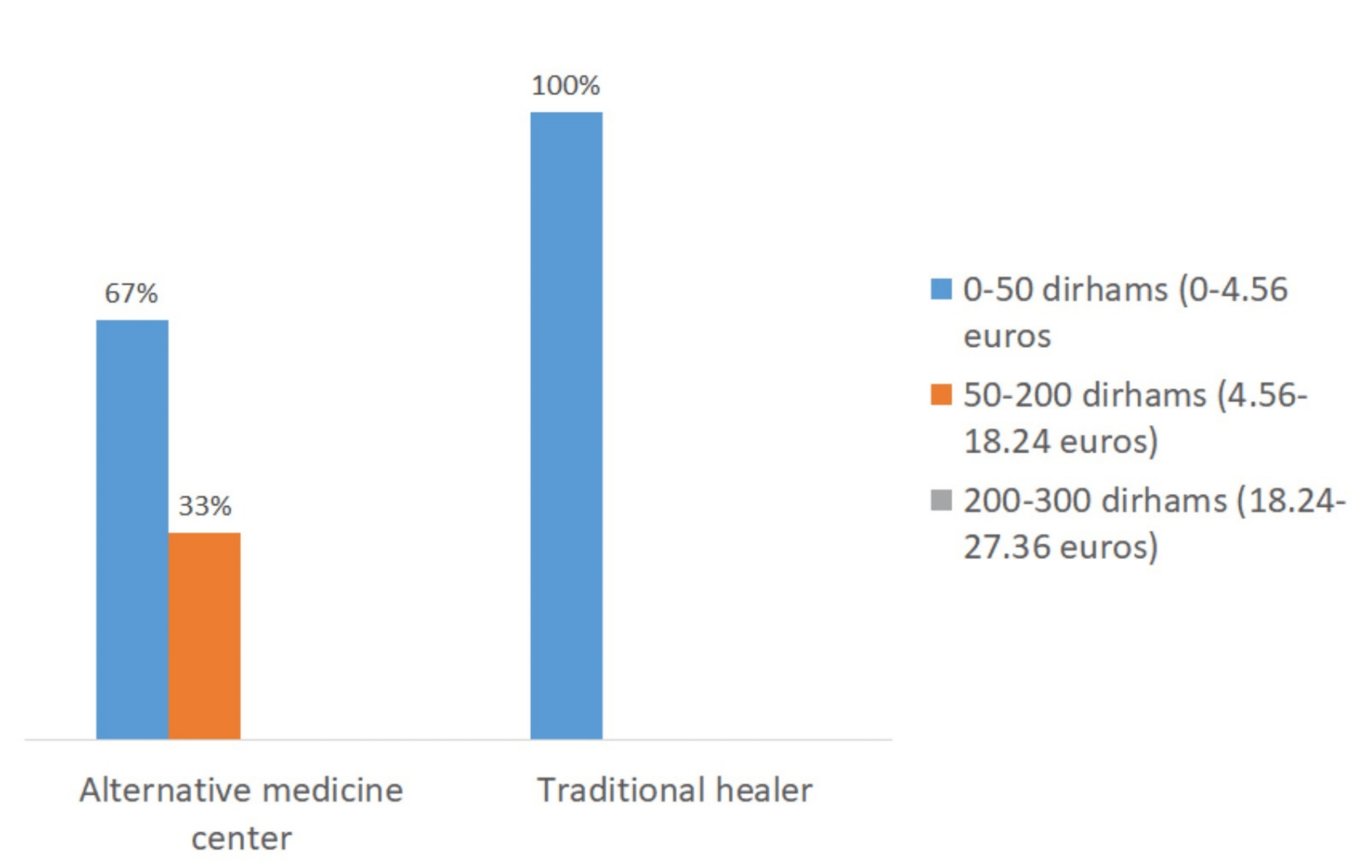
Cost of apitherapy session according to practice location.

**Figure 9:**
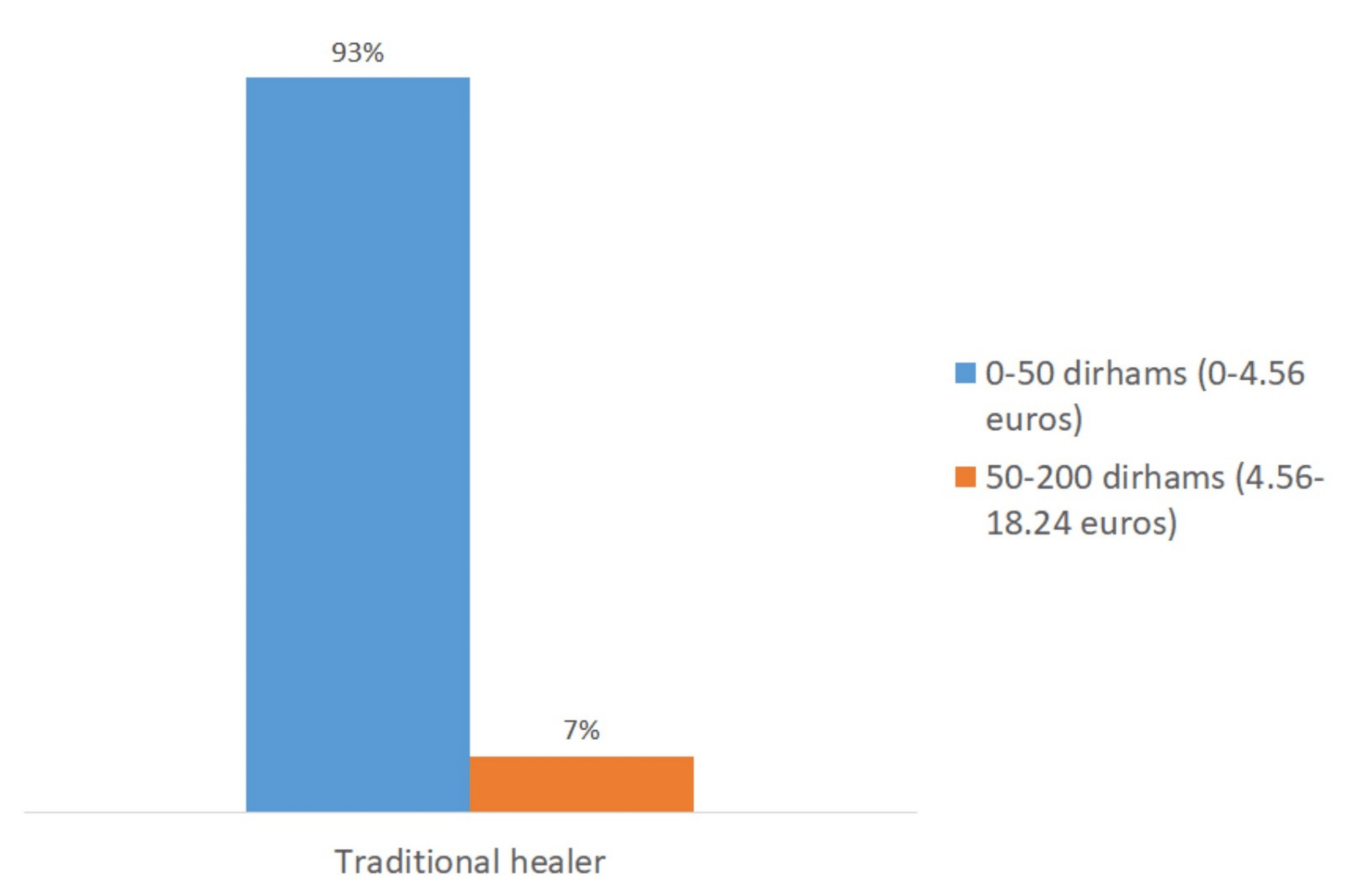
Cost of traditional cautery sessions according to practice location.

The variation in pricing observed for each of these practices was influenced by the location and type of services provided (Figures 7-9). For cupping therapy, 40% of patients paid more than 200 dirhams (equivalent to 18.24 euros) per session when visiting doctors’ offices, whereas 45% of patients consulting traditional healers paid less than 50 dirhams (equivalent to 4.56 euros) per session.

Factors influencing complementary and alternative medicine usage

On univariate analysis, education level was significantly associated with CAM use (p = 0.01) (as can be seen in Table 1). In multivariate analysis, patients with university studies were significantly using fewer CAM methods than their illiterate counterparts within the same age group, gender, income, and type of pathology, with a significant p-value of 0.04 (OR = 0.05, 95% CI = 0.003-0.92). Conversely, the use of CAM was not affected by age, sex, place of residence, monthly individual income, nature of the illness, or the use of biotherapy. In addition to education level, the presence of an inflammatory disease appeared to be significantly related to the practice of cupping therapies (p = 0.04). However, on multivariate analysis, after adjusting for age, sex, monthly income, and level of education, this association was not statistically significant (Table 2).

**Table 2:** Risk factors affecting the use of complementary and alternative medicine by our patients. OR = odds ratio; CI = confidence interval

Variable	OR	CI	P-value
Age	1.01	0.97-1.06	0.39
Gender			
Female	1.39	0.43-4.5	0.57
Education level			
Illiterate	Reference		
Primary school	1.83	0.47-7.17	0.38
Secondary school	1.44	0.27-7.6	0.66
High school	0.65	0.13-3.21	0.6
University studies	0.05	0.003-0.92	0.04
Presence of an inflammatory rheumatic disease	2	0.71-5.65	0.18

## Discussion

In our study, nearly half of the patients (46%) used at least one of the CAM practices considered, namely, cupping therapy, apitherapy, and traditional cautery. Nationally, a 2012 Moroccan study reported that only 12% of RA patients used cupping therapy [6], whereas our study showed a prevalence of 47%, suggesting a significant increase over the past decade. Conversely, the prevalence of traditional cautery use decreased from 20.4% in 2012 to 10% in our study.

Our findings align with a prospective cross-sectional study in Saudi Arabia [7], where 44.5% of participants used alternative medicine, despite differences in populations and complementary methods. However, our results differed from those of other studies. For example, a Turkish study by Gözcü et al. involving 95 patients with rheumatic diseases reported a lower CAM usage rate of 32% [8]. It is important to interpret these prevalence rates with caution, as the Turkish study included phytotherapy, dietary supplements, and other non-medicinal methods not covered in our survey. Other studies conducted in Saudi Arabia [9] and Mexico [10,11] involving patients with RA reported higher prevalence rates but included methods beyond our scope.

Cupping therapy was the most commonly used form of CAM among our patients and was perceived as the most effective, despite the subjective nature of this assessment and the fact that the duration of the improvement was not evaluated in our study. Its popularity may stem from perceived fewer adverse effects compared to conventional treatments and other CAM methods. Marketing, especially by practitioners and celebrities on social media, also plays a role. According to the US National Center for Complementary and Integrative Health, while cupping therapy may offer pain relief, the evidence is not robust, and high-quality research is needed to draw definitive conclusions [12].

Cupping therapy has been evaluated in various musculoskeletal pathologies within rheumatology, including cervicalgia, low back pain, and fibromyalgia, in studies conducted in different countries. However, systematic reviews have identified methodological biases that hinder definitive clinical conclusions [13-17]. The most frequent reason for CAM use in our study was pain alleviation. Limited evidence suggests cupping therapy provides short-term pain relief and reduces functional disability in chronic pain patients compared with no treatment. However, this evidence was constrained by clinical heterogeneity and risk of bias [18].

Among patients who had previously used CAM, those with chronic inflammatory rheumatic diseases (RA and AS) had a higher prevalence of cupping therapy use. In a separate study evaluating the prevalence of CAM use in rheumatic diseases, a higher proportion of patients with RA and fibromyalgia were found to be CAM users, while patients with AS generally did not use CAM [19]. While cupping therapy is commonly used for pain relief among patients with chronic inflammatory rheumatic diseases, the evidence supporting its efficacy remains limited and varied. Further high-quality research is necessary to draw definitive conclusions about its benefits for these conditions [20].

Our study revealed that patients with university-level education used CAM less frequently than other patients. This contrasts with the Turkish study by Gözcü et al. [8], where higher education was associated with greater CAM use. Several factors might explain this discrepancy. In our study, lower CAM use among university-educated patients could be due to their greater access to and reliance on conventional medical treatments. Higher education levels often correlate with better health literacy, leading to a preference for evidence-based practices over CAM. Educated patients might also be more skeptical of CAM’s efficacy due to their familiarity with scientific research. Conversely, the Turkish study’s findings might reflect cultural differences or variations in healthcare systems. In some cultures, CAM practices might be more widely accepted and integrated into routine healthcare, regardless of education level. Additionally, individuals with higher education levels in Turkey might have better access to information about CAM and thus be more inclined to explore these options alongside conventional treatments. The differing results highlight the importance of considering cultural, economic, and healthcare system factors when interpreting CAM use across different populations. Understanding these variables can help tailor healthcare strategies to better meet the needs of diverse patient groups.

Nearly half of our patients had a monthly individual income below the minimum wage, which could explain their reliance on alternative medicine services provided by traditional healers and alternative practice centers. This preference is primarily due to the affordability of these services compared to those offered by doctors and physiotherapists. Additionally, the patient population in this study, drawn from consultations and hospitalizations in our public university hospital, predominantly consisted of individuals with low economic status and limited income. This highlights the need for accessible and affordable healthcare options to better serve economically disadvantaged patients.

Our findings indicate that 92% of study participants learned about CAM through family and friends, aligning with similar observations in another study from Saudi Arabia [7]. Considering that CAM methods can have adverse effects, as found in our study, it is crucial to inquire about patients’ CAM use.

In our study, the majority of patients (91%) did not voluntarily disclose their CAM usage to their healthcare providers. In two-thirds of these cases, this was because their doctors had not initiated conversations about CAM practices. This trend of low disclosure is also observed in the Turkish study by Gözcü et al., where only 31.3% of patients informed their physicians about their CAM use. To mitigate potential adverse effects, healthcare providers should proactively ask patients about their CAM use. This can improve patient safety and ensure a more comprehensive approach to their care. Given the high prevalence of CAM use in our study, improving education on the role of CAM and its integration with conventional treatment can enhance patient outcomes and ensure a safer, more informed approach to healthcare.

This study has several strengths. It is the first to focus on the use of cupping therapy, apitherapy, and traditional cautery among patients with rheumatic diseases in Morocco, providing a unique perspective on patient preferences and practices. Additionally, it includes a cost analysis of these CAM methods, offering valuable insights into the financial aspects of these practices. However, the study has limitations. The cross-sectional design restricts our ability to establish causality or monitor changes over time. The single-center nature and the relatively small sample size of 100 patients may limit the generalizability of our findings. Moreover, the study population, predominantly consisting of individuals with low economic status, may not be representative of the broader population of patients with rheumatic diseases in Morocco, as economic factors might influence the preference for CAM methods over conventional treatments due to cost considerations. Despite these limitations, the study provides valuable insights into the prevalence and patterns of CAM use among patients with rheumatic diseases in Morocco. It highlights the need for further research and more comprehensive education for both patients and healthcare providers on the use of CAM. Future studies with larger, more diverse populations and longitudinal designs would be beneficial to further explore these practices and their impacts on patient health outcomes.

## Conclusions

Our study highlighted the significant prevalence of CAM use among Moroccan patients with rheumatic diseases, with cupping therapy being the most common and subjectively perceived as the most effective. It also revealed that patients with higher education levels were less likely to use CAM. To improve awareness and understanding of CAM practices, it is crucial to educate both patients and healthcare providers about the benefits and risks of these methods to prevent unregulated use. Therefore, we recommend implementing educational programs for healthcare providers to discuss CAM with patients and utilizing various communication channels to reach a broader audience. Further research with larger, diverse populations is essential to explore the efficacy, perceived benefits, and risks of CAM practices to guide informed healthcare decisions.
